# Identification of Key Genes and Pathways in Genotoxic Stress Induced Endothelial Dysfunction: Results of Whole Transcriptome Sequencing

**DOI:** 10.3390/biomedicines10092067

**Published:** 2022-08-24

**Authors:** Maxim Sinitsky, Anna Sinitskaya, Daria Shishkova, Alexey Tupikin, Maxim Asanov, Maria Khutornaya, Marsel Kabilov, Anastasia Ponasenko

**Affiliations:** 1 Laboratory of Genome Medicine, Research Institute for Complex Issues of Cardiovascular Diseases, 650002 Kemerovo, Russia; 2 Laboratory for Molecular, Translation and Digital Medicine, Research Institute for Complex Issues of Cardiovascular Diseases, 650002 Kemerovo, Russia; 3 Institute of Chemical Biology and Fundamental Medicine, Siberian Branch of the Russian Academy of Sciences, 630090 Novosibirsk, Russia

**Keywords:** mutagenesis, atherogenesis, endothelial disfunction, genotoxic stress, DNA damage, RNA-seq, transcriptomic signature, bioinformatic analysis, differentially expressed genes, gene ontology enrichment

## Abstract

Atherosclerosis is a leading cause of cardiovascular morbidity and mortality worldwide. Endothelial disfunction underlying the atherogenesis can be triggered by genotoxic stress in endothelial cells. In the presented research whole transcriptome sequencing (RNA-seq) of human coronary artery (HCAEC) and internal thoracic artery (HITAEC) endothelial cells in vitro exposed to 500 ng/mL mitomycin C (treatment group) or 0.9% NaCl (control group) was performed. Resulting to bioinformatic analysis, 56 upregulated differentially expressed genes (DEGs) and 6 downregulated DEGs with absolute fold change ≥ 2 and FDR *p*-value < 0.05 were selected in HCAEC exposed to mitomycin C compared to the control group; in HITAEC only one upregulated DEG was found. According to Gene Ontology enrichment analysis, DEGs in HCAEC were classified into 25 functional groups of biological processes, while in HITAEC we found no statistically significant (FDR *p*-value < 0.05) groups. The four largest groups containing more than 50% DEGs (“signal transduction”, “response to stimulus”, “biological regulation”, and “regulation of biological process”) were identified. Finally, candidate DEGs and pathways underlying the genotoxic stress induced endothelial disfunction have been discovered that could improve our understanding of fundamental basis of atherogenesis and help to justification of genotoxic stress as a novel risk factor for atherosclerosis.

## 1. Introduction

According to the World Health Organization statistics, atherosclerosis with a different clinical manifestation including ischemic heart disease, ischemic stroke and peripheral arterial disease is a leading cause of cardiovascular morbidity and mortality worldwide [1,2]. Atherosclerosis is a chronic inflammatory disease of large and medium-sized arteries characterized by the accumulation of modified lipids, inflammatory cells, and cell debris in atherosclerotic plaques within the vascular wall [3]. The acute rapture of plaques leads to local thrombosis followed by partial or total occlusion of affected artery [4]. It is known that endothelial disfunction underlying the atherogenesis and defined as a loss of functionality in terms of anti-inflammatory, anti-thrombotic, and vasodilatory abilities of endothelial cells [5] can be triggered by the various risk factors including low or non-laminar shear stress, metabolic, and chemical stress (diabetes mellitus, high serum cholesterol, or effects of cigarette smoke) [6]. Recently it was reported that genotoxic stress defined as a situation that initiates DNA damage compromising the cell’s genomic integrity leading to replication and transcription arrest [7] in endothelial cells in vitro exposed to alkylating mutagen mitomycin C (MMC) followed by DNA alkylation and DNA crosslinking can be considered as another risk factor for endothelial disfunction [8,9]. At the same time, the molecular mechanisms of genotoxic stress induced endothelial disfunction are still not clear [10]. Discovering the molecular pathways underlying this process could improve our understanding of atherogenesis and help to justification of genotoxic stress as a novel risk factor for atherosclerosis. Nowadays, this problem is very important for the modern biomedicines given the increasing genotoxic load on the human organism from both environmental and anthropogenic sources.

High-throughput whole transcriptome sequencing (RNA-seq) is a modern genomics technology allowing access a gene expression signature by determining the primary structure of transcripts. In particular, RNA-seq can be used to detection genes characterized by a differential transcription in different cell types that allows identifying any signaling pathways determining their phenotypes [11].

The presented study is aimed to the identification of key genes and molecular pathways involved in the genotoxic stress induced endothelial disfunction resulting to the in vitro exposure of endothelial cell to MMC. 

## 2. Materials and Methods

### 2.1. Cell Culture

Cryovials containing 5 × 10^5^ primary Human Coronary Artery Endothelial Cells (HCAEC) and Human Internal Thoracic Endothelial Cells (HITAEC) (Cell Applications Inc., San Diego, CA, USA) were thawed; cells were transferred into fibronectin-coated 75 cm^2^ culture flasks containing 15 mL of Human MesoEndo Cell Growth Medium (Cell Applications Inc., San Diego, CA, USA) and cultured in the MCO-18AIC CO_2_ Incubator (Sanyo Electric Co., Ltd., Japan) at 37 °C, 5% CO_2_ in humidified conditions. After 80% confluency was achieved, cells were trypsinized and reseeded into new fibronectin-coated 75 cm^2^ culture flasks (eight flasks for HCAEC and eight flasks for HITAEC) and cultured until 80% confluency was achieved. Then cells were refed with 15 mL of fresh Human MesoEndo Cell Growth Medium (Cell Applications Inc., San Diego, CA, USA) containing 500 ng/mL MMC (AppliChem, Spain, CAS No. 50-07-7) (treatment group, four culture flasks) or 0.9% NaCl (control group, four culture flasks) followed by 6 h incubation at 37 °C, 5% CO_2_ and humidified conditions. Then the cell growth medium was removed, cells were washed twice by ice-cold phosphate buffered saline (PBS) and refed with another 15 mL of additive-free Human MesoEndo Cell Growth Medium (Cell Applications Inc., San Diego, CA, USA) followed by 24 h incubation. All manipulations with HCAEC and HITAEC were started at the same time and performed in parallel.

### 2.2. Whole Transcriptome Sequencing (RNA-Seq)

Whole transcriptome sequencing was performed in the SB RAS Genomics Core Facility, Institute of Chemical Biology and Fundamental Medicine (Novosibirsk, Russia). Cell growth medium was removed; cells were washed by ice-cold PBS and immediately lysed with 2 mL of TRIzol^TM^ Reagent (Invitrogen, Waltham, MA, USA) followed by total RNA isolation using PureLink^TM^ RNA Micro Kit (Life Technologies, Waltham, CA, USA) and DNase treatment (On-Column DNase I Digestion Set, Sigma-Aldrich, Burlington, MA, USA). RNA integrity (RIN) index was accessed using RNA 6000 Pico Kit (Agilent Technologies, Santa Clara, CA, USA) on the 2100 Bioanalyzer system (Agilent Technologies, Santa Clara, CA, USA); RNA quantification was performed using NanoDrop^TM^ 2000 Spectrophotometer (ThermoFisher, Waltham, CA, USA) and Qubit^TM^ 4 Fluorometer (Invitrogen, Waltham, MA, USA).

For the 2–5 μg of isolated total RNA, mRNA was purified using NEBNext^®^ Poly(A) mRNA Magnetic Isolation Module (NEB, Ipswich, MA, USA) followed by DNA library preparation using MGIEasy RNA Directional Library Prep Set (MGI Tech Co., Ltd., China). Each mRNA sample was labeled by their individual barcode. Quality of DNA libraries was analyzed using Agilent High Sensitivity DNA Kit (Agilent Technologies, Santa Clara, CA, USA) on the 2100 Bioanalyzer system (Agilent Technologies, Santa Clara, CA, USA). DNA libraries were quantified by quantitative polymerase chain reaction (qPCR) on the CFX96 Touch Real-Time PCR Detection System (Bio-Rad Laboratories Inc., Hercules, CA, USA). DNA libraries were equimolarly mixed and sequenced on MGISEQ-2000 (MGI Tech Co., Ltd., China) with the paired end reads of 130 + 70 bp.

For each studied groups, four biological replicates were analyzed. All manipulations were performed in accordance with the manufactures’ protocols.

### 2.3. Bioinformatical Analysis

RNA-seq results were analyzed using the CLC Genomic Workbench 21.0.5 (Qiagen, Germany). The raw data obtained through the RNA-seq were filtered by quality (QV > 20) and length (>15), adapters were removed. Read mapping to the human genome (hg38 with Ensembl annotation v.38.105) was performed according to the following parameters: similarity fraction = 0.8, length fraction = 0.8, mismatch cost = 2, insertion cost = 3, and deletion cost = 3.

The RNA-seq read data were submitted to the GenBank under the study accession PRJNA872257.

### 2.4. Identification of Differentially Expressed Genes

Differentially expressed genes (DEGs) were identified using multifactorial statistical analysis based on the negative binomial regression by the CLC Genomic Workbench 21.0.5 (Qiagen, Germany). Statistically significant DEGs were selected based on the following cut-of criteria: absolute fold change ≥ 2 and FDR (false discovery rate) *p*-value < 0.05.

### 2.5. Gene Ontology Enrichment Analysis

Gene Ontology (GO) enrichment analysis against the GO terms of the biological processes category was performed using the CLC Genomic Workbench 21.0.5 (Qiagen, Germany) with the application of FDR correction. The FDR *p*-value cut-off criteria was assigned as 0.05.

## 3. Results

### 3.1. Identification of DEGs Involved in The Genotoxic Stress Induced Endothelial Disfunction

**Figure 1:**
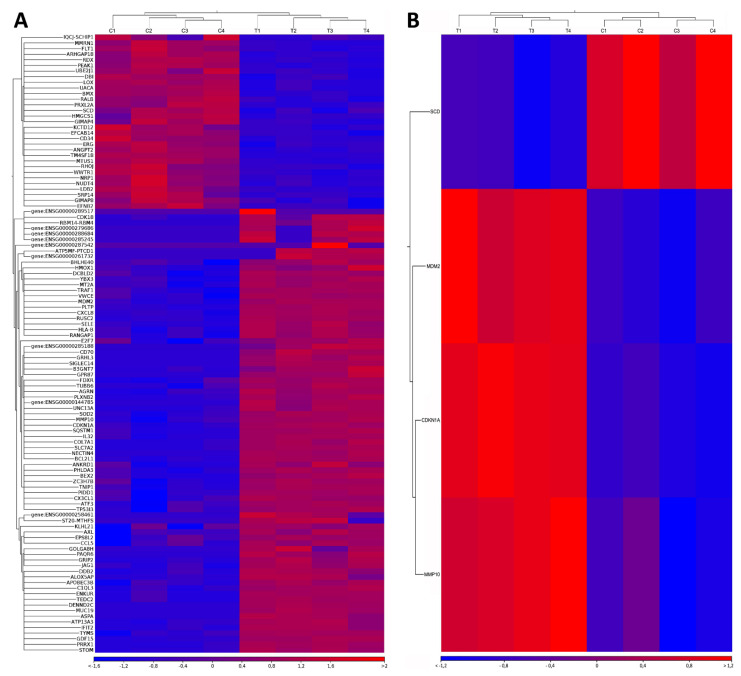
Heatmaps of differentially expressed genes (DEGs) identified in (**A**) Human Coronary Artery Endothelial Cells (HCAEC) and (**B**) Human Internal Thoracic Endothelial Cells (HITAEC) exposed to mitomycin C (MMC) in comparison with the non-exposed control (only those having FDR *p*-value < 0.05 are presented in the heatmaps). Top panel—the results of hierarchical sample clustering (C1, C2, C3, and C4—the control group, T1, T2, T3, and T4—the treatment group). Left panel—the results of hierarchical DEGs clustering (DEGs labeling based on Ensembl annotation). Bottom panel—the absolute fold change.

Resulting to bioinformatical analysis, 110 and 4 DEGs were totally identified in HCAEC (Figure 1A) and HITAEC (Figure 1B) exposed to MMC in comparison with the non-exposed control, respectively (FDR *p*-value < 0.05). After applying the cut-off criterion (absolute fold change ≥ 2), 56 upregulated and 6 downregulated DEGs including 8 novel transcripts (*ENSG00000144785*, *ENSG00000258461*, *ENSG00000261732*, *ENSG00000279686*, *ENSG00000285188*, *ENSG00000285245*, *ENSG00000287542* and *ENSG00000288684*) were selected from the total DEGs in HCAEC (Table 1). At the same time, in HITAEC only one upregulated DEG was found after applying the cut-off criterion (Table 1). Thus, overwhelming majority of identified DEGs were upregulated (90.3% in HCAEC and 100% in HITAEC), while only 9.7% of DEGs in HCAEC were downregulated.

### 3.2. Results of GO Enrichment Analysis

**Figure 2:**
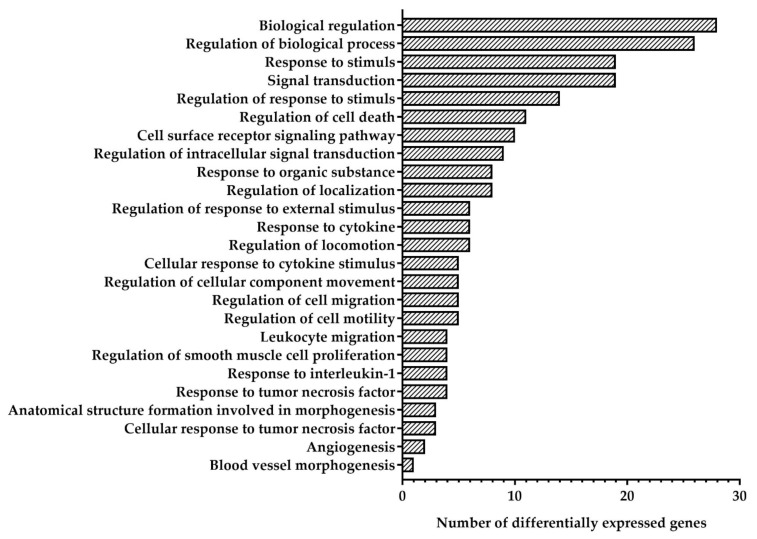
Quantity of differentially expressed genes (DEGs) identified in Human Coronary Artery Endothelial Cells (HCAEC) exposed to mitomycin C (MMC) in comparison with the non-exposed control in functional groups of biological processes according to Gene Ontology enrichment analysis.

The GO is a controlled vocabulary containing more than 38,000 precise defined phrases called GO terms and describing the molecular functions of gene products, the biological processes in which those functions involved and their cellular locations [12]. Resulting to GO enrichment analysis, DEGs in HCAEC were classified into 25 functional groups of biological processes (Figure 2). Four largest groups containing more than 50% DEGs were identified: “signal transduction”, “response to stimulus”, “biological regulation”, and “regulation of biological processes”. Analysis of DEGs distribution between identified functional groups showed that “regulation of intracellular signal transduction”, “response to organic substance”, “response to cytokine”, “cellular response to cytokine stimulus”, “leucocyte migration”, “regulation of smooth muscle cell proliferation”, “response to interleukin-1”, “response to tumor necrosis factor”, “anatomical structure formation involved in morphogenesis”, “cellular response to tumor necrosis factor”, and “angiogenesis” were identified only among upregulated DEGs, while group “blood vessel morphogenesis” contains only downregulated DEG (Table 2). Groups “biological regulation”, regulation of biological processes”, “response to stimulus”, “signal transduction”, “regulation of response to stimulus”, “regulation of cell death”, “cell surface receptor signaling pathway”, “regulation of localization”, “regulation of response to external stimulus”, “regulation of locomotion”, “regulation of cellular component movement”, “regulation of cell migration”, and “regulation of cell motility” were identified both in upregulated and downregulated DEGs (Table 2). Genes *GIMAP8*, *NECTIN4*, *SIGLEC14*, *RBM14-RBM4*, *MUC19*, *COL7A1*, *CD70*, *PAQR6*, *ST20-MTHFS*, *GRP87*, *DENND2C*, *GRHL3*, *UNC13A*, *ENKUR*, *IFIT2*, *ATP13A3*, *GRIP2*, *B3GNT7*, *CDK18*, *TEDC2*, *BEX2*, *APOBEC3B*, *MMP10,* and *RUSC2* were no classified into any functional groups.

## 4. Discussion

Nowadays, the study of endothelial physiology including mechanisms of endothelial dysfunction is one of the most relevant topics in cardiovascular biology and biomedicine. In the last 20 years, the proportion of relevant publications have increased from 5.99% in 2001 to 10.83% in 2021; pathophysiological mechanisms underlying endothelial dysfunction (atherogenic effects of turbulent flow, endothelial-to-mesenchymal transition, impaired endothelial mechanotransduction, etc.) have been described [13]. At the same time, recently it was reported that genotoxic stress can also trigger the pathological activation of endothelium and the formation of a proatherosclerotic phenotype by endothelial cells. Our previously published results demonstrated that endothelial cells in vitro exposed to 500 ng/mL alkylating mutagen MMC can trigger severe genotoxic but not cytotoxic effects in endothelial cells [8] accompanied with the increased mRNA level of genes involved in the proinflammatory activation of endothelium (endothelial proinflammatory cytokines *IL6* and *CXCL8*, endothelial cell receptors to leukocytes *VCAM1*, *ICAM1,* and *SELE*), impaired endothelial mechanotransduction (*KLF4*) and endothelial-to-mesenchymal transition (*SNAI1*, *SNAI2,* and *TWIST1*) [8,9] and suggested as molecular markers of endothelial disfunction [13].

In the presented study, we obtained the novel data about key genes and molecular pathways involved in the genotoxic stress induced endothelial dysfunction in HCAEC and HITAEC. Due to the specific physiological and hydrodynamic characteristics, various vessels are characterized by differential susceptibility to atherogenesis [14]. Human coronary artery is most often affected by atherosclerotic process due to reduced nitric oxide bioavailability, activity of endothelial nitric oxide synthase (eNOS) [15] and its cofactor tetrahydrobiopterin involved in the regulation of both endothelial and inducible nitric oxide synthases, cellular redox signaling and vascular inflammation [16,17,18,19]. On the other hand, atherosclerotic lesions of human internal thoracic artery are diagnosed quite rarely [20]. In the presented, we identified 56 upregulated and 6 downregulated DEGs in HCAEC exposed to MMC in comparison to non-exposed control and only one upregulated DEG in HITAEC (*MDM2*) that was consistent with the literature data on the increased susceptibility of human coronary artery to atherogenesis.

MDM2 is a negative regulator of the tumor suppressor p53 [21] playing a critical role in endothelial dysfunction. It was shown, that endothelial p53 prevents eNOS phosphorylation and impairs endothelium-dependent vasodilatation; its overexpression in Mdm2/Mdm4 deficient mice leads to impaired endothelial function [22,23,24]. Moreover, the p53 plays the important role in the DNA damage response by halting the cell cycle and facilitating the DNA damage repair [25,26]. On the other hand, MDM2 is a component of the TRIM28/KAP1-ERBB4-MDM2 complex which links growth factor and DNA damage response pathways [27]. According to performed in the presented study bioinformatical analysis, we discovered a number of upregulated DEGs genes involved in the p53 signaling pathway in HCAEC: *TP53I3*—induced by the tumor suppressor p53 and involved in p53-mediated cell death and DNA damage response [28,29,30]; *GPR87*—can be upregulated by p53 and DNA damage in a p53-dependent manner and is essential for p53-dependent cell survival in response to DNA damage [31]; *PIDD1*—a component of the DNA damage/stress response pathway functioning downstream of p53/TP53 that can induce p53-dependent apoptosis [32,33,34]; *PHLDA3*—p53/TP53-regulated inhibitor of AKT1 activity via preventing AKT1-binding to the cellular membrane lipids [35,36]; *CDKN1A*—p53/TP53-mediated inhibitor of cellular proliferation through prevention phosphorylation of cyclin-dependent kinase substrate in response to genotoxic stress [37]; *E2F7*—upregulated by p53/TP53 following genotoxic stress and acts as a downstream effector of p53/TP53-dependent repression by mediating repression of indirect p53/TP53 target genes involved in DNA replication [38]. In addition, *E2F7* can promote angiogenesis by acting as a transcription activator: associates with HIF1A, recognizes and binds the VEGFA promoter, and finally activates expression of the VEGFA gene [39]. Thus, MMC treatment of endothelial cells leads DNA damage induced activation of the p53 signaling pathway that leads to impaired endothelial function.

Overexpression of *TYMS* (thymidylate synthase) in HCAEC exposed to MMC may be a compensatory response to the genotoxic stress. It was found that TYMS maintaining the thymidine-5-prime monophosphate pool critical for DNA replication and DNA damage repair via catalyzing the deoxyuridylate transforming to deoxythymidylate using 10-methylenetetrahydrofolate as a cofactor [40].

Adhesion of mononuclear blood fraction to the surface of endothelial cells triggered by the increased expression of cell adhesion molecules is one of the main pathways underlying endothelial dysfunction [41]. In the presented research, we discovered the overexpression of genes, encoding cell adhesion molecules, including the verified marker of endothelial dysfunction *SELE* [13], as well as novel *NECTIN4*. Nectins acting at the cell-cell junctions in a calcium-independent manner belong to the IgSF family of cell adhesion molecules [42]. In human, the *NECTIN4* expression can be found in the placental and embryonic tissues, as well as in lung, breast, pancreatic, ovarian, and head/neck cancers [43]. Interaction of nectin-4 with the cytoskeletal protein actin through the formation of nectin–afadin (F-actin binding protein) complex, leads to the activation of signaling pathways involved in intercellular communication. The nectin–afadin complex can trigger the PI3K/AKT signaling pathway, followed by activation of NF-κB pathway that enhances cell survival and inhibits apoptosis [44,45]. Nectin–afadin–cadherin complex plays an important role in the producing adherens and tight junctions, which regulate cell growth, differentiation, adhesion, migration, and apoptosis [46]. Nectin-4 is also associated with various cell proliferation and angiogenesis related proteins [47].

Proinflammatory activation of endothelial cells in response to genotoxic stress is confirmed by upregulation of proinflammatory cytokine *CD70* from TNF ligand family, proinflammatory chemokines *CCL5*, *CXCL8*, and *CX3CL1,* and ligand of the transforming growth factor-beta superfamily *GDF15* acting as a pleiotropic cytokine involved in the stress response program of cells after cellular injury [48]. It was shown that serum blood level of GDF15 is increased in patients with peripheral artery diseases [49,50]; their protective role against atherogenesis was described [51]. Recently, it was suggested that GDF15 could be produced by endothelial cells during a vascular stress to attenuate endothelial cells loss of function via improves endothelial colony forming cells proliferation, migration, and NO production [52].

AXL receptor tyrosine kinase involving in transducing signals from the extracellular matrix into the cytoplasm by binding growth factor GAS6 can regulate various physiological processes including cell survival, proliferation, migration, and differentiation [53]. It is known that GAS6/AXL signaling plays a role in the protection of endothelial cells from apoptosis through serine-threonine kinase (AKT) phosphorylation, NF-κB activation, increased BCL2 (BCL2 apoptosis regulator) protein expression, and a reduction in caspase 3 activation [54].

Transcription factor *ATF3* has been shown to be upregulated in response to different extracellular signals and tissue/cell injury in tissue/cell-specific manners. It was suggested that *ATF3* can be induced in response to cellular stress [55] including genotoxic stress triggered by ionizing radiation, methyl methanesulfonate, and ultraviolet light [56] through the JNK/SAPK signaling pathway. Extracellular signals induce the JNK/SAPK pathway followed by overexpressing of MEKK (component of the JNK/SAPK pathway) that leads to the increased CAT report driven by an ATF3 promoter fragment. The ATF3 promoter contains potential binding sites for ATF2 and c-Jun (transcription factors phosphorylated and activated by this pathway). Finally, overexpressing of ATF2 and c-Jun together increases the ATF3 promoter activity [55,57].

*PLTP* encodes a protein involved in the transfer of phospholipids and free cholesterol from low-density lipoproteins (LDL) and very low-density lipoproteins (VLDL) into high-density lipoproteins (HDL) and the exchange of phospholipids between LDL and VLDL themselves [58]. In mouse models, PLTP deficiency reduces VLDL and LDL levels and attenuates atherosclerosis, while PLTP overexpression exerts an opposite effect [59,60]. Thus, lipoprotein metabolism is another pathway involved in the genotoxic stress induced endothelial dysfunction.

Cyclin-dependent kinases (CDKs) are the group of molecules involved in the DNA damage response signaling pathway and coordinated cell cycle checkpoints [61,62]. It was shown that downregulation of CDK18 leads to the decrease in RAD17 and RAD9 chromatin retention in response to genotoxic stress followed by the increased level of endogenous DNA damage and chromosomal abnormalities. CDK18-depleted cells are characterized by suppressed kinetics of replication fork and reduces ATR kinase signaling in response to replication stress [62]. In the presented research, the overexpression of *CDK18* may indicate the activation of repair of DNA damage induced by MMC.

Basic helix–loop–helix family member e40 (BHLHE40) is involved in the regulation of inflammatory response [63]—it can promote macrophage proinflammatory gene expression and functions through upregulation of *HIF1α* in macrophages [64]. The possibility was shown of BHLHE40 to repress *IL10* [65] expression and increase expression of *CXCL12* [66].

Endothelial cell migration capacity and apoptosis is required for maintenance of endothelial function. *GRHL3* is underlying this process via induction of AKT and eNOS phosphorylation independent of vascular endothelial growth factor. GRHL3 promotes endothelial cells migration and inhibits apoptosis (probably by caspase 3 blocking) in endothelial cells in the eNOS-dependent manner [67].

Another gene involved in the apoptosis regulation, *IFIT2*, can promote cell apoptosis via BCL2-dependent mitochondrial pathways [68] and also positively regulate the proinflammatory chemokine *CXCL10* expression [69]. Moreover, *IFIT2* modulates the stability of cytokine mRNA by binding directly to RNA with adenylate-uridylate-rich RNAs [70].

*FDXR* transferring electrons from NADPH to mitochondrial cytochrome P450 enzymes [71] is involved in a number of pathways, including the p53 pathway [72] and reactive oxygen species related apoptosis [73,74]; the upregulation of *FDXR* can be considered as a universal response to DNA damage induced both chemical (anticancer drugs) and physical (ionizing radiation) genotoxic factors [75].

The regeneration of damaged endothelial monolayer can be ensured by endothelial progenitor cells expressing markers of endothelial differentiation (CD34, VE-cadherin and von Willebrand factor) [76]. *VWCE*, a member of the von Willebrand factor (VWF) gene family (Von Willebrand factor C and EGF domains) [77] can modulate activity of VWF and, finally, endothelial differentiation.

*DCBLD2* firstly identified in human coronary artery promotes VEGF-induced proliferation of endothelial cells and angiogenesis and negatively regulates tyrosine phosphatase PTP1B, TC-PTP, and VE-cadherin [78].

A number of genes can be only indirectly involved in MMC induced endothelial dysfunction. So, *PAQR8* is involved in the regulation MAPK signaling pathway [79] associated with the cell proliferation, differentiation, migration, senescence, and apoptosis [80]; *BEX2*, *APOBEC3B,* and *MMP10*—in inflammatory activation of endothelial cells through the NF-κB pathway regulation [81,82,83,84]; *ENKUR*—in PI3K/AKT signaling pathway [85]; *ATP5MF-PTCD1* and *DENND2C* can regulate structure and functional activity of nucleic acids. The role of genes *ST20-MTHFS*, *COL7A1*, *MUC19*, *ATP13A3*, *UNC13A*, *RUSC2*, *GRIP2,* and *EPS8L2* in the context of genotoxic stress induced endothelial dysfunction are not completely clear and require further investigations.

A number of the DEGs detected in the presented research were downregulated in response to genotoxic stress in HCAEC. Hydroxy-3-methylglutaryl-CoA synthase 1 (*HMGCS1*) is the gene encoding a key enzyme in the mevalonate pathway of cholesterol synthesis [86]. In addition, HMGCS1 can promote cell proliferation [87], enhance the integrated stress response (ISR) and interact with the endoplasmic reticulum stress transduction protein kinase [88]. In experiments, downregulation of *HMGCS1* in HUVECs leads to the impaired proliferation and migration of these cells [89].

*EFNB2* encoding ephrin B2 is one of the most important genes involved in angiogenesis. Ephrin B2 regulates the interaction of vascular endothelial growth factor (VEGF) with its receptors (FLT1, KDR, and FLT4) and its co-receptors, NRPs [90], and finally stimulates proliferation and migration of endothelial cells, cell degradation, remodeling of the extracellular matrix, and angiogenesis [91]. Another angiogenesis related gene, *BMX*, plays a critical role in TNF-induced angiogenesis, and implicated in the signaling of two the most important for angiogenesis receptors TEK and FLT1 [92]. LDB2 regulates activity of DDL4 (delta-like ligand 4) associated with endothelial sprouting and vascular remodeling relevant in physiologic and pathologic angiogenesis by binding to its promoter and formation of multimeric complex consisting of LMO2, TAL1, and GATA2. LDB2 also mediates VEGF-induced DLL4 expression in endothelial cells. Reduction or overexpression of *LDB2* in endothelial cells decreased or increased DLL4 expression [93,94,95]. Thus, MMC treatment of leads to downregulation of angiogenesis related genes resulting to impaired angiogenesis.

*IQCJ-SCHIP1* and *GIMAP8* can be suggested as a novel genes requiring further investigation in the context of genotoxic stress induced endothelial dysfunction.

## 5. Conclusions

Resulting to RNA-seq followed by bioinformatical analysis we discovered that HCAEC are characterized by the increased susceptibility genotoxic stress induced endothelial dysfunction compared to HITAEC. Only one upregulated gene involved in the p53 signaling pathway was detected in HITAEC. On the contrary, genotoxic stress induced endothelial dysfunction in HCAEC is mainly associated with the p53, GAS6/AXL, JNK/SAPK, PI3K/AKT, and DNA damage response signaling pathways, inflammatory activation of endothelial cells, inflammatory response regulation, endothelial migration and differentiation, apoptosis, adhesion of mononuclear blood fractions to the plasma membrane of endothelial cells, and oxidative stress response. At the same time, the downregulation of genes involved in the angiogenesis was shown. Thus, we obtained novel data about the fundamental basis of genotoxic stress-induced endothelial dysfunction and identified the key genes and pathways involved in this process.

It should be noted that the obtained results are based only on in vitro modeling of genotoxic stress in endothelial cells; in vivo modeling of genotoxic stress induced endothelial disfunction in physiological conditions is required to extrapolate the described pathways to the human organism.

## Figures and Tables

**Table 1:** The differentially expressed genes (DEGs) identified in Human Coronary Artery Endothelial Cells (HCAEC) and Human Internal Thoracic Endothelial Cells (HITAEC) exposed to mitomycin C (MMC) in comparison with the non-exposed control after applying the cut-off criterion (FDR *p*-value < 0.05, absolute fold change ≥ 2).

HCAEC	HITAEC
**Upregulated genes**	
*NECTIN4* (1175.6-fold), *ENSG00000288684* (999.7-fold), *SIGLEC14* (728.6-fold), *ENSG00000285188* (697.5-fold), *ENSG00000285245* (683.8-fold), *ENSG00000144785* (654.5-fold), *RBM14-RBM4* (647.1-fold), *MUC19* (564.9-fold), *ENSG00000287542* (542.6-fold), *ATP5MF-PTCD1* (493.5-fold), *ENSG00000261732* (467.4-fold), *COL7A1* (431.6-fold), *CD70* (384.7-fold), *PAQR6* (361.5-fold), *ENSG00000279686* (357-fold), *ENSG00000258461* (353.4-fold), *ST20-MTHFS* (316.3-fold), *GPR87* (307.7-fold), *DENND2C* (279-fold), *GRHL3* (269.2-fold), *UNC13A* (215-fold), *ENKUR* (147.6-fold), *IFIT2* (145.5-fold), *GRIP2* (114-fold), *B3GNT7* (95.6-fold), *CDK18* (93.5-fold), *TEDC2* (82.9-fold), *BEX2* (61.2-fold), *APOBEC3B* (59.3-fold), *CCL5* (18.8-fold), *EPS8L2* (5.8-fold), *PLTP* (5.7-fold), *SLC7A2* (5.1-fold), *ATF3* (3.9-fold), *CXCL8* (3.5-fold), *VWCE* (3.5-fold), *GDF15* (3.3-fold), *KLHL21* (3-fold), *PIDD1* (2.9-fold), *TYMS* (2.8-fold), *CX3CL1* (2.7-fold), *E2F7* (2.7-fold), *FDXR* (2.7-fold), *MDM2* (2.4-fold), *PHLDA3* (2.4-fold), *MMP10* (2.4-fold), *SOD2* (2.3-fold), *SELE* (2.3-fold), *BHLHE40* (2.3-fold), *RUSC2* (2.2-fold), *CDKN1A* (2.2-fold), *DCBLD2* (2.1-fold), *AXL* (2.1-fold), *ATP13A3* (2.1-fold), *MT2A* (2.1-fold), *TP53I3* (2-fold)	*MDM2* (2.3-fold)
**Downregulated genes**	
*IQCJ-SCHIP1* (3-fold), *LDB2* (2.2-fold), *GIMAP8* (2.1-fold), *BMX* (2.1-fold), *EFNB2* (2-fold), *HMGCS1* (2-fold)	None detected

**Table 2:** Distribution of differentially expressed genes (DEGs) identified in Human Coronary Artery Endothelial Cells (HCAEC) exposed to mitomycin C (MMC) in comparison with the non-exposed control between functional groups of biological processes according to Gene Ontology enrichment analysis.

Functional Group (GO Term)	Genes
**Upregulated genes**	
Biological regulation (GO:0065007)	*MDM2*, *GDF15*, *CDKN1A*, *SOD2*, *PLTP*, *TP53I3*, *SELE*, *DCBLD2*, *CX3CL1*, *PHLDA3*, *MT2A*, *TYMS*, *ATP13A3*, *CXCL8*, *FDXR*, *AXL*, *E2F7*, *EPS8L2*, *KLHL21*, *BHLHE40*, *PIDD1*, *ATF3*, *CCL5*
Regulation of biological processes (GO:0050789)	*MDM2*, *GDF15*, *CDKN1A*, *SOD2*, *PLTP*, *TP53I3*, *SELE*, *DCBLD2*, *CX3CL1*, *PHLDA3*, *MT2A*, *TYMS*, *CXCL8*, *AXL*, *E2F7*, *EPS8L2*, *KLHL21*, *BHLHE40*, *PIDD1*, *ATF3*, *CCL5*
Response to stimulus (GO:0050896)	*MDM2*, *CDKN1A*, *SOD2*, *SELE*, *DCBLD2*, *CX3CL1*, *PHLDA3*, *MT2A*, *TYMS*, *VWCE*, *CXCL8*, *AXL*, *E2F7*, *EPS8L2*, *BHLHE40*, *PIDD1*, *ATF3*, *CCL5*
Signal transduction (GO:0007165)	*MDM2*, *GDF15*, *CDKN1A*, *SOD2*, *SELE*, *DCBLD2*, *CX3CL1*, *PHLDA3*, *MT2A*, *CXCL8*, *AXL*, *E2F7*, *EPS8L2*, *BHLHE40*, *PIDD1*, *ATF3*, *CCL5*
Regulation of response to stimulus (GO:0048583)	*MDM2*, *GDF15*, *CDKN1A*, *SOD2*, *SELE*, *CX3CL1*, *PHLDA3*, *CXCL8*, *AXL*, *PIDD1*, *ATF3*, *CCL5*
Regulation of cell death (GO:0010941)	*MDM2*, *GDF15*, *SOD2*, *TP53I3*, *CX3CL1*, *AXL*, *BHLHE40*, *PIDD1*, *ATF3*, *CCL5*
Cell surface receptor signaling pathway (GO:0007166)	*GDF15*, *CDKN1A*, *SOD2*, *CX3CL1*, *MT2A*, *CXCL8*, *AXL*, *BHLHE40*, *CCL5*
Regulation of intracellular signal transduction (GO:1902531)	*MDM2*, *GDF15*, *SOD2*, *CX3CL1*, *PHLDA3*, *AXL*, *EPS8L2*, *PIDD1*, *CCL5*
Response to organic substance (GO:0010033)	*MDM2*, *SELE*, *CX3CL1*, *MT2A*, *TYMS*, *CXCL8*, *AXL*, *CCL5*
Regulation of localization (GO:0032879)	*SOD2*, *PLTP*, *SELE*, *CX3CL1*, *CXCL8*, *AXL*, *CCL5*
Regulation of response to external stimulus (GO:0032101)	*CDKN1A*, *SELE*, *CX3CL1*, *CXCL8*, *CCL5*
Response to cytokine (GO:0034097)	*SELE*, *CX3CL1*, *MT2A*, *CXCL8*, *AXL*, *CCL5*
Regulation of locomotion (GO:0040012)	*SOD2*, *CX3CL1*, *CXCL8*, *CCL5*
Cellular response to cytokine stimulus (GO:0071345)	*CX3CL1*, *MT2A*, *CXCL8*, *AXL*, *CCL5*
Regulation of cellular component movement (GO:0051270)	*SOD2*, *CX3CL1*, *CXCL8*, *CCL5*
Regulation of cell migration (GO:0030334)	*SOD2*, *CX3CL1*, *CXCL8*, *CCL5*
Regulation of cell motility (GO:2000145)	*SOD2*, *CX3CL1*, *CXCL8*, *CCL5*
Leucocyte migration (GO:0050900)	*SELE*, *CX3CL1*, *CXCL8*, *CCL5*
Regulation of smooth muscle cell proliferation (GO:0048660)	*CDKN1A*, *SOD2*, *CX3CL1*, *CCL5*
Response to interleukin-1 (GO:0070555)	*SELE*, *CX3CL1*, *CXCL8*, *CCL5*
Response to tumor necrosis factor (GO:0034612)	*SELE*, *CX3CL1*, *CXCL8*, *CCL5*
Anatomical structure formation involved in morphogenesis (GO:0048446)	*CXCL8*, *E2F7*, *BHLHE40*
Cellular response to tumor necrosis factor (GO:0071356)	*CX3CL1*, *CXCL8*, *CCL5*
Angiogenesis (GO:0001525)	*CXCL8*, *E2F7*
**Downregulated genes**	
Biological regulation (GO:0065007)	*BMX*, *EFNB2*, *HMGSC1*, *LDB2*, *IQCJ-SCHIP1*
Regulation of biological processes (GO:0050789)	*BMX*, *EFNB2*, *HMGSC1*, *LDB2*, *IQCJ-SCHIP1*
Response to stimulus (GO:0050896)	*BMX*
Signal transduction (GO:0007165)	*BMX*, *EFNB2*
Regulation of response to stimulus (GO:0048583)	*BMX*, *EFNB2*
Regulation of cell death (GO:0010941)	*EFNB2*
Cell surface receptor signaling pathway (GO:0007166)	*BMX*
Regulation of localization (GO:0032879)	*LDB2*
Regulation of response to external stimulus (GO:0032101)	*EFNB2*
Regulation of locomotion (GO:0040012)	*EFNB2*, *LDB2*
Regulation of cellular component movement (GO:0051270)	*LDB2*
Regulation of cell migration (GO:0030334)	*LDB2*
Regulation of cell motility (GO:2000145)	*LDB2*
Blood vessel morphogenesis (GO:0048514)	*EFNB2*

## Data Availability

The RNA-seq read data were submitted to the GenBank under the study accession PRJNA872257.
